# *Bacillus* spp. Contamination: A Novel Risk Originated From Animal Feed to Human Food Chains in South-Eastern Bangladesh

**DOI:** 10.3389/fmicb.2021.783103

**Published:** 2022-01-04

**Authors:** Md Atiqul Haque, Fei Wang, Yi Chen, Foysal Hossen, Md Aminul Islam, Md Amzad Hossain, Naila Siddique, Cheng He, Firoz Ahmed

**Affiliations:** ^1^Key Lab of Animal Epidemiology and Zoonoses of Ministry of Agriculture and Rural Affairs, College of Veterinary Medicine, China Agricultural University, Beijing, China; ^2^Department of Microbiology, Faculty of Veterinary and Animal Science, Hajee Mohammad Danesh Science and Technology University, Dinajpur, Bangladesh; ^3^Department of Microbiology, Faculty of Science, Noakhali Science and Technology University, Noakhali, Bangladesh; ^4^National Reference Lab for Poultry Diseases (NRLPD), Animal Sciences Institute (ASI), National Agricultural Research Centre (NARC), Islamabad, Pakistan

**Keywords:** food-borne pathogen, *Bacillus* spp., toxin gene, food chain risk, diarrhea, animal production

## Abstract

The current study provides information on *Bacillus* spp. contamination along with present status in commercially available poultry and animal feeds as well as animal-derived products in Bangladesh. The research has been conducted to determine if animal feed and its components are a source of *Bacillus* spp. contamination in feed and food chain. Out of 180 different feeds, milk, egg, and human stool samples, 218 *Bacillus* spp. were isolated and identified by cultural morphology, microscopic, biochemical, and molecular characteristics where *B. cereus*, *B. subtilis*, *B. amyloliquefaciens*, *B. licheniformis*, *B. thuringiensis*, *B. megaterium*, and *B. coagulans* accounted for 51, 22, 9.1, 5.9, 5, 3.6, and 2.2%, respectively. Regarding the enumeration of total viable count and total *Bacillus* count, correspondingly 67 and 39% samples were found to be contaminated with above 10,000 CFU/g, while highest contamination was 85 and 75% in broiler feed, respectively. The total number of bacteria above the regulatory limits in commercially available feeds indicates a poor compliance with regulation and abuse administration in the Bangladeshi market. Moreover, a hospital-based survey showed that food-borne *Bacillus* spp. contributed to 4.5% human diarrhea cases and 25% food contamination associated with vegetables, rice, RTE food, milk, and egg, accounting for 46, 34, 14, 4, and 2%, respectively. *B. cereus* was the dominant isolate correspondingly accounting for 56 and 51% egg and milk contamination followed by *B. amyloliquefaciens* (32%) and *B. thuringiensis* (12%) in egg and *B. subtilis* (25%), *B. amyloliquefaciens* (12%), *B. thuringiensis* (6.4%), and *B. coagulans* (3.2%) in milk, respectively. Toxin gene profiling of *Bacillus* spp. revealed that *B. cereus* constituted a principal part of virulence, while *B. thuringiensis*, *B. licheniformis*, *B. megaterium*, *B. coagulans*, and *B. subtili*s showed genetic diversity and *B. amyloliquefaciens* had not carried any toxin gene. Detection rate of enterotoxin genes (*nheA*, *nheB*, *nheC*, *cytK*, *hblA*, *hblC*, *hblD*, and *entFM*) showed that 55% isolates carried *nheABC* genes, 80% *entFM*, and 71% *cytK*, whereas only 33% of the isolates contained *hblACD* gene clusters. These virulence genes were posing a threat to human health due to spread across the food and feed chain. Finally, our findings support the hypothesis that *B. cereus* might contribute to clinical diarrhea, gizzard erosion, and lung infection in duck and poultry, and that it contaminates animal-derived foods resulting in toxicity and antibacterial resistance to humans. Therefore, maximal tolerance limits of *Bacillus* spp. and their potential risks to the animal industry are urgently needed to clarify. Moreover, *Bacillus* spp.–induced toxin residual must be altered for human health via food chain transmission.

## Introduction

*Bacillus* spp. are Gram-positive, rod-shaped, motile (flagellated), aerobic or facultative anaerobic, spore- and biofilm-forming bacteria commonly found in nature, isolated from fermented or unfermented food and feed stuffs, that have caught attention as potential probiotics, which have been patented in the form of a wide range of health supplements. Probiotics are claimed to provide health benefits for the hosts and commonly fed to humans, animals, and plants to increase the production efficiency in livestock, poultry, and aquaculture combating gastrointestinal infections and alternatives to antibiotics (Elshaghabee et al., 2017; Cui et al., 2019; Gupta et al., 2019; Haque et al., 2021). *Bacillus* spp. have the ability to produce antimicrobial substances as a probiotic and ensure its stability and vitality in different habitats in comparison with conventional commercial probiotics. *Bacillus* spp., especially *B. toyonensis*, *B. licheniformis*, *B. subtilis*, *B. amyloliquefaciens*, *B. thuringiensis*, *B. mycoides*, *B. coagulans*, *B. clausii*, and *B. pumilus*, have been widely used as probiotics in humans, plants, and animal production (Cui et al., 2019; Gupta et al., 2019; Cao et al., 2020).

While probiotic *Bacillus* strains have been shown to inhibit the growth of pathogenic bacteria (*E. coli*, *Clostridium*, *Streptococcus*, and *Salmonella*), certain species, especially those belonging to the *B. cereus* group, are considered to be unsafe due to the possibility of toxin genes (*nhe*, *hbl*, *cytK1*, *ces*) and antibiotic resistance genes (*tet45*, *erm34*, *aadD2*, *bla_*BCL–*1_*, *cat*_*Bcl*_) being transferred from commercial probiotic products (Zhu et al., 2016; Gupta et al., 2019; Zuo et al., 2020; Deng et al., 2021). Although some countries are developing guidelines to ensure the safety of probiotic products, lack of legal inspection or requirement to demonstrate efficacy may pose a threat of getting *B. cereus* contamination in animal-used probiotics, followed by increasing hazard to food safety. Hence, it is necessary to determine whether *Bacillus* spp. strain is pathogenic or beneficial, as well as their connection to those with generally recognized as safe (GRAS) status. Correspondingly, various toxins must be investigated to ensure its safety before adding into animal feeds and human medications. Furthermore, *B. cereus* was a leading cause of food-borne illness in the United States and the third most common food pathogen in China, which causes two distinct gastrointestinal diseases, emesis and diarrhea, as well as many systemic and local complications including hemorrhagic necroses in the central nervous system, fulminant bacteremia, pneumonia, endophthalmitis, and gas gangrene-like cutaneous symptoms in both immune-deficient and immune-competent individuals (Cui et al., 2019; Li et al., 2020; Deng et al., 2021; Haque et al., 2021). In 2018, *B. cereus* was found in 98 reported outbreaks across EU member States, accounting for 1.9% of all outbreaks, affecting 1,539 people, 111 hospitalizations, and 1 death (Rodrigo et al., 2021). Food-borne *B. cereus* poisoning includes animal-derived products (poultry, meat and meat products, puddings, sauces, milk and milk products, pasteurized liquid eggs, fish, stew) and plant-based foods (rice, potatoes, cereals, grains, spices, vegetables, salads) (Haque et al., 2021). The survival potential of *B. cereus* spores in extreme environment poses a significant risk to food safety and also causes economic losses to the food industry. In addition, feed-borne *B. cereus* contamination worsens extreme diarrhea and malnutrition in poultry by causing gizzard erosion and ulceration syndrome, as well as hemorrhagic inflammation in lungs and immunosuppression co-infection with other pathogens (Zuo et al., 2020; Haque et al., 2021). It can lead to major food safety concerns because the formation of spores, biofilms, and various virulence potentials, like heat-tolerant/labile emetic and/or diarrheal toxins and tissue-destructive enzymes, makes it difficult to fully prevent their presence in food (Haque et al., 2021). A recent study in Bangladesh revealed that residues of antimicrobial used against *B. cereus* and *B. subtilis* were found in 68% of liver and 66% of kidney layers, respectively. More interestingly, 33% animal probiotics carried potential pathogens, including human intestinal *cya* toxin gene in the vicinity of chicken and fish farm in a recent Chinese survey (Li et al., 2020; Hassan et al., 2021). Most *Bacillus* spp. (*B. cereus*, *B. subtilis*, *B. licheniformis*) are antibiotic resistant and transfer antibiotic resistance genes to humans, implying that antibiotic residues may enter consumer food products and the human food chain (Deng et al., 2021). This can result in the emergence of multidrug-resistant bacterial strains, with the possibility of resistance gene transfer to other pathogenic and non-pathogenic bacteria (Islam et al., 2017; Haque et al., 2021). Several research on the prevalence of *Bacillus* spp. in various Bangladeshi foods have been conducted, but the isolate’s ability to produce emetic and enterotoxins has yet to be determined. Our hypothesis for this study is to explore the key information on contamination levels and toxigenic profiles of isolated *Bacillus* spp. in animal feeds, animal-derived products, and human stool that will help us better understand the pathogen’s current status and pathogenic potential in Bangladesh. Our findings will be used to assess the risk and apply food safety measures to ensure food security.

## Materials and Methods

### Sampling Sites, and Collection and Processing of Samples

The current study was conducted in Noakhali district (22.70° N, 91.10° E) of Chittagong division, about 151 km from Dhaka, the capital of Bangladesh. A total of 180 samples were collected during September 2019 to June 2020 from various sources, including layer (*n* = 20), broiler (*n* = 20), duck (*n* = 13), fish (*n* = 15), and cattle (*n* = 20) feed, table egg (*n* = 20), milk (*n* = 20), and human stool (50) in different locations across the district. All these samples were collected from layer farms, broiler farms, duck farms, cattle farms, fish farms, selling outlet stores, and a human hospital (Supplementary Table 1). Aseptic procedures were maintained while collecting these samples, with amounts varying by types: 100 g for feed, 20–30 ml for milk, 1 g for stool, and a whole egg. During sample collection, sterile gloves were used to handle feed, egg, and milk samples from each source. In case of milk samples, spot collection was employed; raw milk was gathered during milking of cow into sterile wide-mouth tubes, corked, and carefully labeled. In case of stool samples, sterile screw cap vials were used to avoid environmental contamination. Each sample was immediately kept in sterile Ziploc plastic bags, transported in an insulated foam box with cold chain (temperature, 4–6°C) to the Microbiology Laboratory, Noakhali Science and Technology University, Noakhali, Bangladesh. Upon arrival, all samples were refrigerated at 4°C until microbiological analysis, which was completed within 24 h after receiving the samples.

### Validated Questionnaires

A cross-sectional survey was conducted to enroll participants for interviews and 38 farms were randomly chosen, including layer (*n* = 15), broiler (*n* = 15), and duck farms (*n* = 8). Data on the various types of farms, the age and populations of birds, the type of housing, feeds, egg production, feed additives and antibiotics, feed source, and clinical history were collected using a questionnaire survey (Supplementary Appendix I). A structured data collection schedule was also used to obtain information on diarrheal patients and types of food consumed (Supplementary Appendix II). After getting informed cell phone consent, the researcher interviewed patients to collect data. The experimental protocols were approved by an Ethical Reviewing Board on Institutional Animal Care and Use Committee at Noakhali Science and Technology University. Our survey of 38 farms revealed that majority of the farmers were small-scale producers of broiler (53%) and duck farms (85%) with a maximum of 2,000 birds, and medium-scale producers of layer farms (73%) with a maximum of 5,000 birds compared with the multiple ages broiler farms (6.6%); 100% of the surveyed layer and duck farms had an all-in all-out breeding system, while 93% of broiler farms had the same raising system (Supplementary Tables 2–4).

### Microbiological Analysis

Microbiological qualities of the samples such as total viable count (TVC) and total *Bacillus* count (TBC) were determined using the method described by USDA/FSIS (Microbiology Laboratory Guidebook [MLG], 2015). All the glassware used in this study were sterilized by autoclaving at 121°C for 15 min and then cooling to 45°C. The system was also used for serial dilution, inoculation and incubation, sub-culture, Gram staining, and identification of isolates. Pure cultures were stored at −80°C in glycerol stocks for further study.

#### Isolation and Identification of Bacterial Isolates

##### Bacteriological Examination

The samples were first cultured into Nutrient Agar (NA) (HiMedia India) for TVC and Mannitol–Egg Yolk–Phenol Red–Polymyxin Agar (MYPA) (HiMedia, India) for TBC. The feed samples were homogenized in peptone water in the beaker as described previously (Gyang et al., 2019) by thoroughly mixing 1 g of each sample in 9 ml of peptone water. In case of table eggs, yolks were treated as described previously (Khan et al., 2016). Briefly, the eggs were soaked in 75% alcohol for 5 min before being sterilized for 5–10 s on a hot flame. The egg yolk was then drained into the sterilized polythene bags and manually homogenized after a small hole was cut on the shell surface. In case of milk, 1 ml of sample was homogenized with 9 ml of sterile buffered peptone water (Oxoid, United Kingdom) using a vortex mixture. In case of stool samples, 1 g of stool is diluted in 9 ml sterile buffered peptone water (Oxoid) and homogenized using a vortex mixture for primary enrichment. Finally, each sample was diluted in phosphate buffered saline (PBS) at a ratio of tenfold (1:10) serial dilution. This was accomplished by adding 1 ml homogenized sample to 9 ml PBS, mixing thoroughly with vortexing and repeating until 10^–6^ dilution was obtained (Abraha et al., 2017). These acceptable dilutions were cultured on NA (HiMedia) and MYPA (HiMedia) media using the spread plate technique (Microbiology Laboratory Guidebook [MLG], 2015) and incubated overnight at 37°C in the incubator. Following incubation, plates with 25–250 colonies were counted and TVC as well as TBC were expressed as colony-forming units per gram of sample (CFU/g).

##### Cultural Characterization and Biochemical Test for Bacillus spp. Identification

From the dilution tubes, 10 μl of each dilution was spot dropped in triplicate onto MYPA (HiMedia) for *Bacillus* spp. isolation. The plates were incubated at 37°C for 24 h and following incubation colonies on the media were studied morphologically, including size, shape, surface texture, edge, elevation, color, and opacity. To identify and validate the isolated colonies, the Gram staining and following biochemical tests were performed: Triple Sugar Iron (TSI) Test, Motility Indole Urease (MIU) Test, Oxidase Test, Catalase Test, Voges Proskauer (VP) Test, Simon Citrate Agar Test, and Starch Hydrolysis Test as described previously (Zhang et al., 2019). For identification and confirmation, all presumptive colonies of *Bacillus* spp. were purified and subjected to morphological and biochemical tests as defined in A Color Atlas of Bacillus Species (Parry et al., 1988).

##### Molecular Identification

Genomic DNA was extracted by boiling method as described previously (Hu et al., 2021). Briefly, positive colonies grown overnight in nutrient broth at 37°C, then 1 ml was taken in Eppendorf tube followed by centrifuging at 13,000 rpm for 10 min and resuspending in 200 μl sterile Milli-Q water (Sigma Aldrich). Then the resuspended bacterial cell suspension was boiled at 100°C for 10 min, placed on ice immediately for 10 min, and finally centrifuged at 10,000 rpm for 10 min to obtain the genomic DNA of each isolate. The concentration of DNA was determined with a Colibri LB-915 Microvolume Spectrophotometer (Berthold Technologies, Germany) and the sample was diluted to a final concentration of approximately 100 ng/μl. Toxin gene profiling and distribution of emetic and enterotoxin gene of *Bacillus* spp. was evaluated by PCR. The targeted genes included non-hemolytic (*nheA*, *nheB*, *nheC*) and hemolytic (*hblA*, *hblC*, *hblD*) complexes, *CytK*, *entFM*, and *Ces*. PCR protocols were performed as previously described (Ehling-Schulz et al., 2006; Sergeev et al., 2006; Table 1). *Bacillus cereus* ATCC 14579 was used as positive control and sterile Milli-Q water (Sigma Aldrich) was used for negative control. The PCR mixture was prepared in a volume of 25 μl, with OneTaqQuick-Load 2 × Master Mix (New England Biolabs Inc., United States), 0.2 μmol L^–1^ final concentration of each primer, and 2.5 μl of prepared DNA template. PCR was performed on T100 Thermal cycler (Bio-Rad, United States). The PCR products were separated on 1.5% agarose gel (MP Biomedicals LLC, United States) using Mini-Sub Cell GT Horizontal Electrophoresis System (Bio-Rad, United States), stained with ethidium bromide, visualized on a UV transilluminator (Gel Doc EZ), and photographed by gel documentation system.

**TABLE 1:** Primers used in this study.

Name	Sequence	T_m_ (°C)	Product size (bp)	References
*nheA*	F = TTTCTATCGGTACTTTAAGTAATGAAATTGTA	63.5	405	Sergeev et al., 2006
	R = AACTGTTTAATGTACTTCAACGTTTGTAAC	63.9		
*nheB*	F = TTATAAAGTAATGGCTCTATCAGCACT	62.2	750	
	R = TACTGCACCACCGATAATTGCAA	61.1		
*nheC*	F = GTTCAGTTGTGAGCAGGAGCTT	62.1	620	
	R = AAACTATTTGTATCTTTCGCCATTCTAT	61.3		
*CytK*	F = GTAACAGATATCGGKCAAAATGCA	60.1	527	
	R = TGTTATATCCRTTAAAGAATACGTTCCA	61.3		
*hblA*	F = CGACGCTATTAACTATTACAACTGCTA	63.7	265	
	R = GTAACAGCATGTGCCCTTGCA	61.3		
*hblC*	F = TATAACAAAGGAAAAGAAATTAACAACTCTA	61.7	641	
	R = CATGACTATTCTCCTTCTTTCGCTAA	63.2		
*hbld*	F = TGCACAAGAAACGACCGCTCA	61.3	987	
	R = ATAATTTGCGCCCATTGTATTCCAT	60.9		
*entFM*	F = AAAGAAATTAATGGACAAACTCAAACTCA	62.0	609	
	R = GTATGTAGCTGGGCCTGTACGT	64.0		
*ces*	F = GGTGACACATTATCATATAAGGTG	60.1	1271	Ehling-Schulz et al., 2006
	R = GTAAGCGAACCTGTCTGTAACAACA	64.2		

### Data Analysis

Data are expressed as mean ± SEM and analyzed using SPSS v.25 software. The one-sample *t*-test and χ^2^ test were applied to determine the cumulative difference among the parameters studied. Statistically significant difference was judged as *p < 0.05 and ^**^p < 0.01.

## Results

### Determination of *Bacillus* spp. in Different Feeds, Milk, Egg, and Human Stool

Frequency of *Bacillus* spp.–positive samples and TVC and TBC of various feeds, eggs, milk, and stool samples are shown in Tables 2, 3, respectively. Positive *Bacillus* spp. were found in 100% of layer feed, broiler feed, egg, and milk, as compared with 80, 73, 70, and 16% in duck feed, fish feed, cattle feed, and human stools, respectively. In terms of TVC, 67% contaminated samples yielded more than 10,000 CFU/g, with the highest and lowest contamination rates being 85 and 55% in broiler feed (*p* < 0.01) and egg, respectively. In terms of TBC, 39% contaminated samples were above 10,000 CFU/g, with the highest and lowest contamination rates being 75 and 5% in broiler feed (*p* < 0.01) and egg, respectively.

**TABLE 2:** Positive *Bacillus* spp. in feed and other samples.

Feed sample	Total	Positive for *Bacillus* spp.	%
Layer feed	20	20	100.0
Broiler feed	20	20	100.0
Duck feed	15	12	80.0
Cattle feed	20	14	70.0
Fish feed	15	11	73.3
Egg	20	20	100.0
Milk	20	20	100.0
Human stool	50	08	16.0
Total	180	117	90.0

*Out of 180 different samples, positive Bacillus spp. was 100% in layer feed, broiler feed, egg, and milk; and 80, 73, 70, and 16% in duck feed, fish feed, cattle feed, and human stool, respectively.*

**TABLE 3:** Enumeration of TVC and TBC in different feed and other samples.

Sample types	No. of samples	TVC (CFU/g)				TBC (CFU/g)				
		10–1,000	1,000–10,000	>10^4^	Mean ± SEM	No count	10–1,000	1,000–10,000	>10^4^	Mean ± SEM
Layer feed	20	1 (5.0)	7 (35.0)	12 (60.0)	2.55 ± 0.135^b^	0 (0.0)	2 (10.0)	5 (25.0)	13 (65.0)	3.55 ± 0.153^c^
Broiler feed	20	0 (0.0)	3 (15.0)	17 (85.5)	2.85 ± 0.082^c^	0 (0.0)	1 (5.0)	4 (20.0)	15 (75.0)	3.70 ± 0.128^c^
Duck feed	15	2 (13.3)	3 (20.0)	10 (66.6)	2.53 ± 0.192^b^	2 (13.3)	1 (6.6)	5 (33.3)	7 (46.6)	3.13 ± 0.274^a^
Cattle feed	20	2 (10.0)	4 (20.0)	14 (70.0)	2.60 ± 0.152^c^	6 (30.0)	1 (5.0)	4 (20.0)	9 (45.0)	2.80 ± 0.296^a^
Fish feed	15	1 (6.6)	4 (26.6)	10 (66.6)	2.60 ± 0.163^b^	3 (20.0)	0 (0.0)	6 (40.0)	6 (40.0)	3.00 ± 0.293^a^
Egg	20	4 (20.0)	5 (25.0)	11 (55.0)	2.35 ± 0.182^a^	12 (60.0)	3 (15.0)	4 (20.0)	1 (5.0)	1.70 ± 0.291^c^
Milk	20	0 (0.0)	4 (20.0)	16 (80.0)	2.80 ± 0.092^c^	0 (0.0)	1 (5.0)	5 (25.0)	14 (70.0)	3.65 ± 0.131^c^
Human stool	50	8 (16.0)	10 (20.0)	32 (64.0)	2.48 ± 0.108^c^	42 (84.0)	0 (0.0)	2 (4.0)	6 (12.0)	1.44 ± 0.146^c^
Total	180	18 (10.0)	40 (2.2)	122 (67.7)	–	65 (36.1)	9 (5.0)	35 (19.4)	71 (39.4)	–

*TVC, total viable count; TBC, total Bacillus count; CFU, colony-forming unit. ^a^Indicates p > 0.05 when compared with the average CFU of TVC and TBC in the same column. ^b^Indicates p < 0.05 when compared with average CFU of TVC and TBC in the same column. ^c^Indicates p < 0.01 when compared with average CFU of TVC and TBC in the same column. The data are expressed as the mean ± SEM.*

Regarding *Bacillus* spp., *B. cereus*, *B. subtilis*, *B. amyloliquefaciens*, *B. licheniformis*, *B. thuringiensis*, *B. megaterium*, and *B. coagulans* were isolated from layer, broiler, duck, cattle and fish feed, egg, milk, and human stool. Out of 218 isolates, 112 strains (51%) of *B. cereus*, 49 strains (22%) of *B. subtilis*, 20 strains (9.1%) of *B. amyloliquefaciens*, 13 strains (5.9%) of *B. licheniformis*, 11 strains (5%) of *B. thuringiensis*, 8 strains (3.6%) of *B. megaterium*, and 5 strains (2.2%) of *B. coagulans* were identified by morphological and biochemical tests (Table 4 and Supplementary Table 5). Obviously, *B. cereus*, *B. subtilis*, and *B. amyloliquefaciens* were dominant distributions in all the tested samples, accounting for 181 isolates (83%) (Table 4).

**TABLE 4:** Positive *Bacillus* spp. isolated in different feed and other samples.

Sample type	Sample no.	Positive species						
		*B. cereus*	*B. subtilis*	*B. amyloliquefaciens*	*B. licheniformis*	*B. thuringiensis*	*B. megaterium*	*B. coagulans*
LF	42	22 (52.3)	12 (28.5)	1 (2.3)	3 (7.1)	2 (4.7)	2 (4.7)	0 (0.0)
BF	37	19 (51.3)	9 (24.3)	2 (5.4)	2 (5.4)	3 (8.1)	1 (2.7)	1 (2.7)
DF	26	14 (53.8)	5 (19.2)	4 (15.3)	1 (3.8)	1 (3.8)	1 (3.8)	0 (0.0)
CF	28	12 (42.8)	7 (25.0)	0 (0.0)	5 (17.8)	0 (0.0)	3 (10.7)	1 (3.5)
FF	19	8 (42.1)	5 (26.3)	1 (5.2)	2 (10.5)	0 (0.0)	1 (5.2)	2 (10.5)
E	25	14 (56.0)	0 (0.0)	8 (32.0)	0 (0.0)	3 (12.0)	0 (0.0)	0 (0.0)
M	31	16 (51.6)	8 (25.8)	4 (12.9)	0 (0.00	2 (6.4)	0 (0.0)	1 (3.2)
HS	10	7 (70.0)	3 (30.0)	0 (0.0)	0 (0.00	0 (0.0)	0 (0.0)	0 (0.0)
Total	218	112 (51.3)	49 (22.4)	20 (9.1)	13 (5.9)	11 (5.0)	8 (3.6)	5 (2.2)

*LF, layer feed; BF, broiler feed; DF, duck feed; CF, cattle feed; FF, fish feed; E, egg; M, milk; HS, human stool. Out of 218 isolates, 51% B. cereus, 22% B. subtilis, 9.1% B. amyloliquefaciens, 5.9% B. licheniformis, 5% B. thuringiensis, 3.6% B. megaterium, and 2.2% B. coagulans were identified. The data are expressed as percentage.*

More interestingly, *Bacillus* spp. from human diarrhea and food contamination (egg and milk) had 4.5 and 25% positivity, respectively. Regarding human diarrhea, *B. cereus* was dominant among the isolates (70%), followed by *B. subtilis* (30%). Likewise, 56% eggs and 51% milk were determined to have *B. cereus* contamination, followed by *B. amyloliquefaciens* (32%) and *B. thuringiensis* (12%) in egg, and *B. subtilis* (25%), *B. amyloliquefaciens* (12%), *B. thuringiensis* (6.4%), and *B. coagulans* (3.2%), respectively, in milk (Table 4).

### Toxin Gene Profiles and Distribution of Emetic and Enterotoxin Genes of Isolated *Bacillus* spp.

**FIGURE 1:**
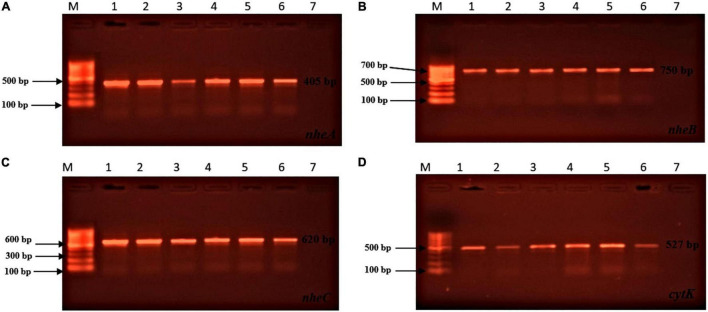
Toxin genes of isolated *Bacillus* spp. by PCR test. **(A)** (*nheA* gene, L1: 100 bp marker, L2: positive control, L3: sample 1, L4: sample 2, L5: sample 3, L6: sample 4, L7: sample 5, L8: negative control); **(B)** (*nheB* gene, L1: 100 bp marker, L2: positive control, L3: sample 1, L4: sample 2, L5: sample 3, L6: sample 4, L7: sample 5, L8: negative control); **(C)** (*nheC* gene, L1: 100 bp marker, L2: positive control, L3: sample 1, L4: sample 2, L5: sample 3, L6: sample 4, L7: sample 5, L8: negative control); **(D)** (*cytK* gene, L1: 100 bp marker, L2: positive control, L3: sample 1, L4: sample 2, L5: sample 3, L6: sample 4, L7: sample 5, L8: negative control).

**FIGURE 2:**
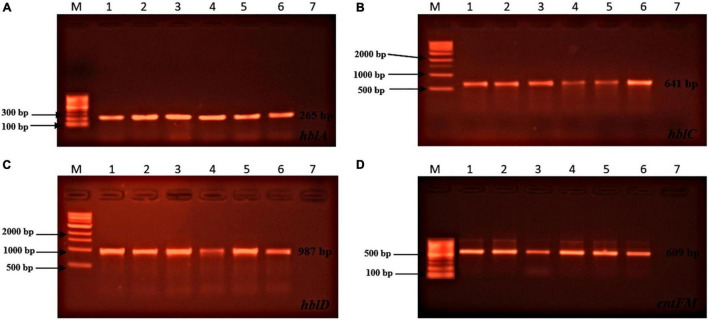
Toxin genes of isolated *Bacillus* spp. by PCR test (continued). **(A)** (*hblA* gene, L1: 100 bp marker, L2: positive control, L3: sample 1, L4: sample 2, L5: sample 3, L6: sample 4, L7: sample 5, L8: negative control); **(B)** (*hblC* gene, L1: 1 kb marker, L2: positive control, L3: sample 1, L4: sample 2, L5: sample 3, L6: sample 4, L7: sample 5, L8: negative control); **(C)** (*hblD* gene, L1: 1 kb marker, L2: positive control, L3: sample 1, L4: sample 2, L5: sample 3, L6: sample 4, L7: sample 5, L8: negative control); **(D)** (*entFM* gene, L1: 100 bp marker, L2: positive control, L3: sample 1, L4: sample 2, L5: sample 3, L6: sample 4, L7: sample 5, L8: negative control).

**FIGURE 3:**
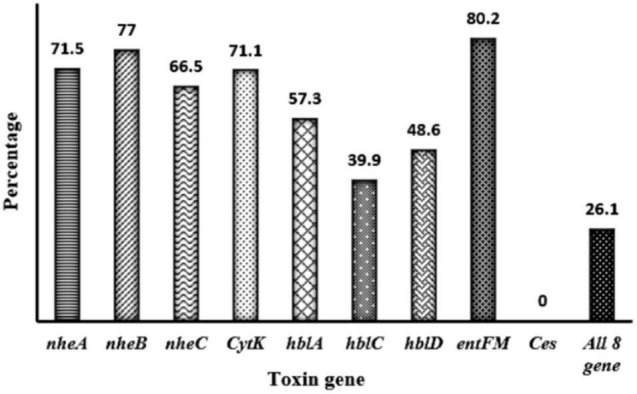
Distribution of toxin gene of *Bacillus* spp. isolated from animal feed, animal-derived products, and human stool in Bangladesh. The number at the top of the bars represents the positive rate of the corresponding toxin genes. “All eight genes” presents the strains containing all the detected toxin genes.

**FIGURE 4:**
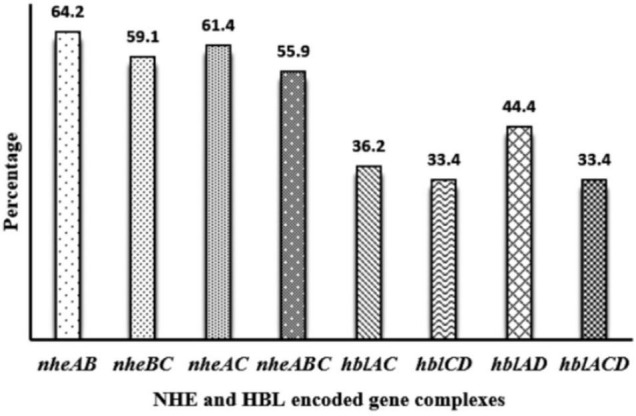
Distribution of *nhe*-based gene and *hbl*-based gene cluster of *Bacillus* spp. isolated from animal feed, animal-derived products, and human stool in Bangladesh. The number at the top of the bars represents the positive rate of the corresponding toxin gene clusters.

Toxin genes of isolated *Bacillus* spp. by PCR test as indicated in Figures 1A–D, 2A–D showed the specific amplified target band. The toxin gene profiles of *Bacillus* spp. were classified into 24 different types of isolates suggesting genetic diversity (Table 5). The distribution of virulence genes among 218 *Bacillus* spp. isolates is shown in Supplementary Tables 6–7 and Figures 3, 4. Positivity of *entFM* (80%) gene was higher among the enterotoxin genes than other genes of *nheB* (77%), *nheA* (71%), *cytK* (71%), *nheC* (66%), *hblA* (57%), *hblD* (48%), and *hblC* (39%). Regarding *nhe*-based gene clusters, 64% of the strains harbored enterotoxigenic gene *nheAB* compared with 61% *nheAC*, 59% *nheBC*, and 55% *nheABC*, whereas 17% of the isolated did not possess *nhe*-based genes. For the *hbl*-based gene clusters, only 44% of the isolates possessed *hblAD*, 36% *hblAC*, 33% *hblCD*, and 33% *hblACD*, whereas 35% of the isolates did not possess an *hbl*-based gene. All the *Bacillus* spp. isolates were negative for emetic toxin gene *Ces*. Distribution of virulence gene in the *B. cereus* isolates obtained from animal feed correspondingly possessed 73, 56, 34, 72, 54, 36, 46, and 84% for *nheA*, *nheB*, *nheC*, *cytK*, *hblA*, *hblC*, *hblD*, and *entFM* genes. Furthermore, *B. cereus* carried virulence genes with the rate of 63, 36, 26, 66, 46, 40, 50, and 80% in animal-derived foods and 71, 42, 28, 85, 57, 42, 28, and 71% in human diarrheal case for *nheA*, *nheB*, *nheC*, *cytK*, *hblA*, *hblC*, *hblD*, and *entFM*, respectively. In contrast, two other dominant isolates, *B. subtilis* and *B. amyloliquefaciens*, had no major toxin gene; only 33% strains of *B. subtilis* carried *hblD* in human diarrheal cases and 25, 28, and 33% of *nheA* were identified from the animal feed, animal-derived food, and human diarrheal cases, respectively (Supplementary Table 8).

**TABLE 5:** Toxin gene profiling of *Bacillus* spp. Isolates.

Profile	Toxin gene									LF (*n* = 42)	BF (*n* = 37)	DF (*n* = 26)	CF (*n* = 28)	FF (*n* = 19)	E (*n* = 25)	M (*n* = 31)	HS (*n* = 10)	Total (*n* = 218)
	*nheA*	*nheB*	*nheC*	*CytK*	*hblA*	*hblC*	*hblD*	*entFM*	*ces*									
1	+	+	+	+	+	+	+	+	−	13	15	9	7	5	2	1	5	57 (26.1)
2	+	+	+	+	+	+	+	−	−	2	3				1	2		8 (3.6)
3	+	+	+	+	+	+	−	+	−		1	1	2	2				6 (2.7)
4	+	+	−	+	+	+	+	+	−	1	1						1	3 (1.3)
5	+	−	+	+	+	+	+	+	−				1	1	1	2		5 (2.2)
6	+	+	+	+	+	−	−	+	−			1	1	1				3 (1.3)
7	+	+	+	+	−	−	+	+	−	1	1		1		1			4 (1.8)
8	+	+	−	+	+	−	+	+	−			2	5	1		7		15 (6.8)
9	+	+	+	+	−	+	−	+	−	3		3			2			8 (3.6)
10	+	+	+	+	+	−	−	−	−		1					2	1	4 (1.8)
11	+	+	+	+	−	−	−	+	−	11	7		5	2	1	1		27 (12.3)
12	+	−	+	+	+	−	+	−	−			1		2		2		5 (2.2)
13	−	+	+	+	+	−	−	+	−	1	2		1		2	1		7 (3.2)
14	+	+	+	−	−	−	−	+	−			3				2		5 (2.2)
15	+	−	+	+	−	−	−	+	−				1			1		2 (0.9)
16	−	+	−	−	+	−	+	+	−	1	2						1	4 (1.8)
17	−	−	+	−	−	−	+	+	−			2		1				3 (1.3)
18	−	−	−	−	+	−	−	+	−			1	1		3	1		6 (2.7)
19	−	−	−	−	−	−	+	+	−					1		2		3 (1.3)
20	−	+	−	−	−	−	−	+	−						2			2 (0.9)
21	+	−	−	−	−	−	−	−	−					1	3			4 (1.8)
22	−	+	−	−	−	−	−	−	−	2	1	1	2	1				7 (3.2)
23	−	−	−	−	−	−	−	+	−	2	1	1			2	3	2	11 (5.0)
24	−	−	−	−	−	−	−	−	−	5	2	1	1	1	5	4		19 (8.7)

*LF, layer feed; BF, broiler feed; DF, duck feed; CF, cattle feed; FF, fish feed; E, egg; M, milk; HS, human stool; +, positive; −, negative.*

### Validated Questionnaire Between Production Metric and *Bacillus* spp. Contamination

Regarding *Bacillus* spp. contamination in broiler and duck production parameters, at least 20% farms were affected with diarrhea (*p* < 0.05) with moderate decrease in growth and feed conversion rate. Moreover, mortality (*p* < 0.05) was recorded to reach 20% in broiler and 12% in duck farm and more than 2% delay of slaughtering time was observed (Table 6). In contrast, *Bacillus* spp. contamination was associated with layer production parameters; 13% farms were affected with diarrhea (*p* < 0.01), small decrease in egg production, and low-quality eggs (*p* < 0.05), as well as a slight increase in culling rate (*p* < 0.05) and mortality (*p* < 0.01) in 13% farms of over 2% (Table 7). Regarding hospital-based survey, clinical diarrhea cases due to *B. cereus* (70%) and *B. subtilis* (30%) infection were linked to consuming diets while vegetables, rice, RTE food, milk, and eggs were accounted for 46, 34, 14, 4, and 2%, respectively (Tables 4, 8).

**TABLE 6:** Association between *Bacillus* spp. contamination and meat bird’s production parameters.

Characteristics	Broiler farm (*n* = 15)			Duck farm (*n* = 8)		
	No of farms	%	Mean ± SEM	No of farms	%	Mean ± SEM
**Clinical diarrhea**						
Present	3	20.0	1.80 ± 0.107^b^	2	25.0	1.75 ± 0.164^a^
Absent	12	80.0		6	75.0	
**Growing speed**						
Optimum	9	60.0	1.40 ± 0.131^a^	5	62.5	1.38 ± 0.183^a^
Decrease	6	40.0		3	37.5	
**Feed conversion**						
Optimum	10	66.6	1.33 ± 0.126^a^	6	75.0	1.25 ± 0.164^a^
Decrease	5	33.3		2	25.0	
**Slaughtering time**						
Normal	11	73.3	1.27 ± 0.118^a^	5	62.5	1.38 ± 0.183^a^
Delay	4	26.6		3	37.5	
**Mortality**						
<2%	12	80.0	1.20 ± 0.107^b^	7	87.5	1.00 ± 0.000^b^
>2%	3	20.0		1	12.5	

*^a^Indicates p > 0.05 when compared with the production parameters of bird in the same column. ^b^Indicates p < 0.05 when compared with production parameters of bird in the same column. The data are expressed as the mean ± SEM.*

**TABLE 7:** Association between *Bacillus* spp. contamination and layer’s production parameters.

Characteristics	Layer farm (*n* = 15)		Mean ± SEM
	No of farms	%	
**Clinical diarrhea**			
Present	2	13.3	1.87 ± 0.091^c^
Absent	13	86.6	
**Egg production**			
Optimum	10	66.6	1.33 ± 0.126^a^
Decrease	5	33.3	
**Egg quality**			
Excellent	2	13.3	2.07 ± 0.153^b^
Good	10	66.6	
Poor	3	20.0	
**Culling rate**			
Normal	12	80.0	1.20 ± 0.107^b^
Increase	3	20.0	
**Mortality**			
<2%	13	86.6	1.13 ± 0.091*^c^*
>2%	2	13.3	

*^a^Indicates p > 0.05 when compared with the production parameter of bird in the same column. ^b^Indicates p < 0.05 when compared with the production parameter of bird in the same column. ^c^Indicates p < 0.01 when compared with the production parameter in the same column. The data are expressed as the mean ± SEM.*

**TABLE 8:** Association between human diarrhea cases and daily consuming diets.

Daily consuming diet	Clinical diarrhea cases (*n* = 50)		Cooking type
	No of people	%	
Vegetables	23	46	Raw, half and fully cooked
Rice	17	34	Fully cooked
RTE food	7	14	Fully cooked
Milk	2	4	Half cooked
Egg	1	2	Half and fully cooked

*According to a hospital-based survey, clinical diarrhea cases due to Bacillus spp. infection linked to contaminated daily consuming diet contributing vegetables, rice, RTE food, milk, and egg were 46, 34, 14, 4, and 2%, respectively. The data are expressed as percentage.*

### Postmortem Findings of Clinical Case of Duck Farms and Isolation of *Bacillus* spp. From Eggs and Human Diarrheal Case

Necropsy revealed mortality on two affected ducks during field investigation. Postmortem findings showed ulceration on gizzard and inflammation and hemorrhagic lesions on lungs caused by the bacteria (Supplementary Figures 1, 2). On culture from the gizzard and lungs, *B. cereus* was found to be responsible for gizzard erosion and lung inflammation (Supplementary Figure 3). *Bacillus* spp. were isolated from table eggs and human stools (Supplementary Figures 4, 5).

## Discussion

Food-borne diseases are likely one of the most serious public health problems in Bangladesh concerning food safety challenges and their associated economic and social costs (Chowdhury et al., 2021). This is the first study of the *Bacillus* spp. contamination levels in poultry (layer, broiler, and duck) feed, cattle feed, animal products (milk and eggs), and human stool in Bangladesh. In the present study, 51% of all samples were *B. cereus* contamination, which was more or less the same as those of previous surveys in other types of foods elsewhere, i.e., 20–48% in Korea, 50% in China, 52% in Spain, and 57% in Mexico (Yu et al., 2019). Moreover, 55% isolates harbored *nheABC* and 33% isolates carried *hblACD* gene clusters, respectively, while 80% isolates harbored *entFM* gene and 71% isolates possessed *cytK* gene. Our findings confirmed that feed-borne *B. cereus* might cause clinical diarrhea in duck and poultry by producing diverse toxins, contributing to lower productivity, toxin residual in eggs and milk, and the threat to public health via feed chain. On the other hand, the maximal tolerance limit of *B. cereus* in most of food is set up by the regulatory authorities, 10^2^– < 10^3^ (Food Standard Australia and New Zealand), <10^4^CFU/g (European Food Safety Authority), <10^3^– ≤ 10^5^ (Health Protection Agency United Kingdom, Center for Food Safety, Hong Kong, China) as mentioned in Haque et al. (2021). The widespread distribution of *B. cereus* including thermoresistant spores attributable to the ingestion of foods contaminated with this pathogen containing >10^4^ CFU/g is not acceptable and unsafe for human consumption. *Bacillus* spp. are found in vegetables, rice, animal products (milk and eggs), and RTE food as a result of a variety of factors, including soil or air contact during cultivation, transportation, and distribution; spore or cell transfer in the dairy farm via feed and bedding material; raw milk contamination during the milking procedure, from within or exterior of the udder, and the surface of the milk handling utensils; and subsequent temperature abuse during shipment. The inclusion of cracked and contaminated eggs, cross-contamination, and poor hygienic conditions may result in the occurrence of *B. cereus* in these foods (Soni et al., 2016; Islam et al., 2017; Yu et al., 2019; Haque et al., 2021). Hence, it is of great concern that food safety criteria for *B. cereus* were not met in 70% of the positive milk samples and 5% of egg, thus signaling a potentially dangerous risk. More interestingly, 46% human diarrheal cases were attributed to consumption of vegetable diet contaminated with *B. cereus*, which is similar to findings in several countries (Yu et al., 2019). *B. cereus* spore adhesion and resistance to external environment can lead to biofilm formation, where vegetative cells may survive on vegetables, when slow cooling and prolonged storage at room temperature have been documented to enable the spores to germinate and develop in cooked rice (Soni et al., 2016; Antequera-Gómez et al., 2021). RTE foods are highly susceptible to contamination during handling, direct contact with food-contact surfaces, and dirty cutting utensils (Valero et al., 2016).

Diarrhea caused by *B. cereus* is attributed to different enterotoxins produced by the strains in the small intestine including HBL, NHE, and CytK. In this study, eight enterotoxin genes and the presence of *entFM* gene was highest (80%), which was in agreement with other reports (Yu et al., 2019; Zhao et al., 2020; Hu et al., 2021; Kong et al., 2021; Sornchuer and Tiengtip, 2021). The frequency of *nheABC* (55%) and *hblACD* (33%) gene clusters in our study was lower than those reports from different foodstuffs in Thailand, Korea, and China. The frequency of *cytK* (71%) was higher than that observed on market food in Thailand (68%), China (45%), Italy (41%), and Tunisia (37%) but lower than that of the pasteurized milk (73%), meat and meat products (82%), and vegetables (87%) in China and milk products (75%) in Ghana (Owusu-Kwarteng et al., 2017; Gdoura-Ben Amor et al., 2019; Proroga et al., 2019; Yu et al., 2019; Hu et al., 2021; Kong et al., 2021; Sornchuer and Tiengtip, 2021). *Bacillus* spp. have been identified on a variety of substrates, including soil, plant-based foods, corn- and soybean-based fermented feedstuffs for livestock and poultry, and fermented functional food for human consumption harboring the blend of *Bacillus* spp. (Elshaghabee et al., 2017).

The distribution of enterotoxin genes in *B. cereus* isolated from different feed, animal-derived products, and human stools suggested that food poisoning by the diarrheal toxin-producing strains cannot be neglected and new probiotic candidate of *B. cereus* group as food and feed additive must be demonstrated with safety evaluation of no toxic potentials. In addition to *B. cereus*, *B. subtilis*, *B. amyloliquefaciens*, *B. licheniformis*, *B. thuringiensis*, *B. megaterium*, and *B. coagulans* have all been linked to food safety issues (Haque et al., 2021). In Bangladesh, approximately 94% of poultry farmers use antibiotics indiscriminately in their farms as a growth promoter, treatment, and prophylaxis, and they seem to be more cautious about the potential costs of meat and egg production (Hosain et al., 2021). Moreover, the use of antibacterial agents in veterinary practice is not strictly controlled, and farmers abuse antibiotics to control poultry disease. In our survey, at least 20, 60, and 12% of farms were confirmed to abuse antibiotics for medication, prophylaxis, and both purposes without withdrawal time, respectively (Supplementary Tables 2–4). *Bacillus* spp. contaminated in animal products can act as reservoir for toxic and virulent genes, increasing the likelihood of developing resistant bacteria in humans. Considering the overall situations like *B. cereus* and *Bacillus* spp. mediated contaminations and spreading of toxin gens followed by aberrant antibiotic uses and mounting resistance, immediate integrated measures are required for the developing world. Contamination of probiotic-borne *Bacillus* spp. can be regulated by specific fermentation parameters such as sampling material composition, subsequent culture processes, fermentation properties, post-operation techniques, and utilization of bacterial peptide bacteriocins with a broad range of antibacterial action against *B. cereus* (Haque et al., 2021). Despite the fact that the Bangladesh Food Safety Authority (BFSA) has recently released food safety regulations and guidelines, there is still much work to be done in terms of raising awareness and putting the animal feeds and food products of animal origin safety standards into practice, securing food safety.

## Conclusion

To summarize, this is the first study in Bangladesh to investigate the quantity of *Bacillus* spp. contamination and toxigenic potentials persistent in animal feed, animal-derived food, and human stool. By detecting and profiling toxin genes as well as completing validated questionnaire surveys, this study assesses the sanitary risk potential of *Bacillus* spp. strains. The findings revealed that this feed-borne *Bacillus* spp. group collection has significant toxigenic potentials, contaminating animal feed (poultry, duck, cattle, fish), animal products (egg, milk), and daily food (vegetables, rice, and RTE food). Humans consume vegetative cells and spores of *Bacillus* spp. on a regular basis via fermented meals and raw vegetables, resulting in diarrhea. Therefore, further research could be allotted to evaluating risk factors, potential pathogenesis, and antibiotic resistance genes of feed-borne *Bacillus* spp. to ensure sustainable animal production and public health. Finally, it emphasizes the importance of prevention and control strategies such as (1) routine checkup both in animal feedstuffs and human food, and (2) R&D test method that allows for rapid screening and species identification.

## Data Availability Statement

All datasets generated for this study were included in the article/Supplementary Material, further inquiries can be directed to the corresponding author/s.

## Ethics Statement

The studies involving human participants were reviewed and approved by the Ethical Reviewing Board at Noakhali Science and Technology University. Written informed consent for participation was not required for this study in accordance with the national legislation and the institutional requirements. The animal study was reviewed and approved by Ethical Reviewing Board at Noakhali Science and Technology University on Institutional Animal Care and Use Committee (ICAUC).

## Author Contributions

CH, FA, and MHa: conceptualization and methodology. MHa, FH, MI, and MHo: perform the experiments. CH and FA: supervise the project. MHa: data curation and writing – original draft. FW and YC: resources. CH, FA, and NS: writing, review, and editing. All authors contributed to the article and approved the submitted version.

## Conflict of Interest

The authors declare that the research was conducted in the absence of any commercial or financial relationships that could be construed as a potential conflict of interest.

## Publisher’s Note

All claims expressed in this article are solely those of the authors and do not necessarily represent those of their affiliated organizations, or those of the publisher, the editors and the reviewers. Any product that may be evaluated in this article, or claim that may be made by its manufacturer, is not guaranteed or endorsed by the publisher.
