# Supplementary material for: Bacillus spp. Contamination: A Novel Risk Originated From Animal Feed to Human Food Chains in South-Eastern Bangladesh
Source: Front Microbiol. 2022 Jan 4;12:783103. doi: 10.3389/fmicb.2021.783103 (PMC8764408; doi:10.3389/fmicb.2021.783103)
Supplement: Supplementary file 1 [file Data_Sheet_1.PDF]

Appendix-I

Department of Microbiology
Faculty of Science
Noakhali Science and Technology University

Survey Questionnaire

1. Name of Farm:

Sample ID:

Owner & Address:

2. Poultry species

Broiler	Layer	Duck	Others

Chicken breed:

Breeding system:

All-in All-out	Multi-age

Age of Bird:

Number of Birds:

Number of House:

3. Types of feed

Starter	Grower	Finisher	Homemade feed

Feed additives

Probiotic	Fermented	Herbal	Others

- If it is yes, please tell the dose, duration

4. Clinical Information: diarrhea, poor performance, increasing mortality, low antibody response

Diarrhea due to probiotic / feed additive

Absent	Present

Body weight gain

Poor	Good	Excellent

Mortality

Yes	No

- Postmortem observation

Gizzard ulceration

Present	Absent	Other

❖ For layer

5. Egg production performance

Initial	Current

For examples: egg production in peak period, lasting months; Regarding egg quality, blood-stain eggs, soft-egg, color fading egg, broken eggs

Egg type

Normal	Abnormal

Percentage of blood-stain eggs, soft-egg, color fading egg, broken eggs

6. Control measures

Antibiotic Use

Now	Last 3 months

Follow withdrawal period

Yes	No

Purpose

Therapeutic	Prophylactic	Both	Others

Control measures: (1) antibiotic types, dose, treating duration; (2) Withdrawal of probiotics; (3) Culling flock due to new profit

----
Signature of the investigator

Date: ……………………………

Note: If necessary Bengali version of the questionnaire will be used while interviewing farm owner.
