# Supplementary material for: Bacillus spp. Contamination: A Novel Risk Originated From Animal Feed to Human Food Chains in South-Eastern Bangladesh
Source: Front Microbiol. 2022 Jan 4;12:783103. doi: 10.3389/fmicb.2021.783103 (PMC8764408; doi:10.3389/fmicb.2021.783103)
Supplement: Supplementary file 2 [file Data_Sheet_2.PDF]

Appendix-II

Department of Microbiology
Faculty of Science
Noakhali Science and Technology University

Data Collection Schedule

1. **Name of the patient:** Serial no. ☐

2. **Address:** **Contact no.........................**

3. **Demographic variables:**
   a) **Age:** ☐ **Years**
   b) **b) Sex: Male** ☐ **Female** ☐
   c) **Education: Illiterate** ☐ **primary** ☐ **secondary** ☐ **SSC** ☐ **HSC** ☐ **Graduate** ☐
   d) **Occupation:** **monthly income** ☐ BDT
   e) **Religion: Islam** ☐ **Hinduism** ☐ **Christianity** ☐ **others** ☐
   f) **Residence: Rural** ☐ **Urban** ☐ **Semi urban** ☐ **Slum**
   g) **Socioeconomic status: Poor** ☐ **Lower Middle class** ☐ **Upper middle class** ☐ **Rich** ☐

4. **Clinical history**
   a) **Duration of diarrhea** ☐ **days**
   b) **Nature of stool: Loose watery** ☐ **Rice watery** ☐ **Mucoid/bloody** ☐
   c) **Stool color:**
   d) **Vomiting: Yes** ☐ **No** ☐
   e) **Dehydration: Yes** ☐ **No** ☐
   f) **Fever: Yes** ☐ **No** ☐
   g) **Body temperature:**
   h) **Abdominal cramp: Yes** ☐ **No** ☐
   i) **Abdominal pain: Yes** ☐ **No** ☐
   j) **Others:**
   k) **Food history: Vegetable** ☐ **Rice** ☐ **RE food** ☐

5. **Antibiotic use:**
   a) **Name of antibiotic**
   b) **Route: Oral** ☐ **Injection** ☐
   c) **Dose:**
   d) **Antibiotic took before:**

Signature of Investigator

Date: ……….........................

Note: If necessary Bengali version of the questionnaire will be used while interviewing patients.
