# Supplementary material for: Bacillus spp. Contamination: A Novel Risk Originated From Animal Feed to Human Food Chains in South-Eastern Bangladesh
Source: Front Microbiol. 2022 Jan 4;12:783103. doi: 10.3389/fmicb.2021.783103 (PMC8764408; doi:10.3389/fmicb.2021.783103)
Supplement: Supplementary file 3 [file Data_Sheet_3.PDF]

Supplemental Table 1. Sample collection site and source

Sample type	Number of samples		Total
Layer feed	Layer farm<br>15	Feed seller<br>5	20
Broiler feed	Broiler farm<br>15	Feed seller<br>5	20
Duck feed	Duck farm<br>8	Feed seller<br>7	15
Cattle feed	Dairy farm<br>10	Feed seller<br>10	20
Fish feed	Fish farm<br>5	Feed seller<br>10	15
Egg	Outlet market<br>20		20
Milk	Dairy farm<br>20		20
Human stool	Prime Hospital Limited<br>50		50
		Grand Total	180

Supplementary Table 2. General characteristics of Layer farms in Noakhali region of Bangladesh

Characteristics	No of farms	%	Mean ± SEM
Layer farm (n=15)			
No. of birds			
500-1000	2	13.3	
1000-5000	9	60.0	2.13±0.165[a]
>5000	4	26.6	
Breeding system			
All-in All out	15	100	1.00±0.000
Multi-age	0	0.0	
Types of feed			
Starter	2	13.3	
Grower	5	33.3	2.47±0.215[a]
Finisher	7	46.6	
Homemade	1	6.6	
Use of feed additive			
Yes	9	60.0	1.40±0.131[a]
No	6	40.0	
Presence of diarrhea			
Yes	2	13.3	1.87±0.091[c]
No	13	86.6	
Birds health status			
Excellent	10	66.6	
Good	5	33.3	1.33±0.126[a]
Poor	0	0.0	
Egg production performance			
Not yet	2	13.3	
Normal	10	66.6	2.07±0.153[b]
Decrease	3	20.0	
Egg type			
Normal	13	86.6	1.13±0.091[c]
Abnormal	2	13.3	
Mortality			
<1%	4	26.6	
1-2%	9	60.0	1.87±0.165[a]
>2%	2	13.3	
Antibiotic use			
Therapeutic purpose	3	20.0	
Prophylactic purpose	10	66.6	1.93±0.153[b]
Both	2	13.3	
Follow withdrawal period			
Yes	10	66.6	1.29±0.125[a]
No	5	33.3	

[a] indicated $p>0.05$ when compared with the characteristics in the same column, [b] indicated $p<0.05$ when compared with the characteristics parameter in the same column, [c] indicated $p<0.01$ when compared with the characteristics in the same column. SEM= Standard error of mean.

Supplementary Table 3. General characteristics of Broiler farms in Noakhali region of Bangladesh

Characteristics	No of farms	%	Mean ± SEM
Broiler farm (n=15)			
No. of birds			
500-1000	3	20.0	
1000-2000	5	33.3	2.27 ±0.206[a]
>2000	7	46.6	
Breeding system			
All-in All out	14	93.3	1.07±0.067[c]
Multi-age	1	6.6	
Types of feed			
Starter	3	20.0	
Grower	8	53.3	2.07±0.182[a]
Finisher	4	26.6	
Homemade	0	0.0	
Use of feed additive			
Yes	5	33.3	1.67±0.126[a]
No	10	66.6	
Presence of diarrhea			
Yes	3	20.0	1.80±0.107[b]
No	11	73.3	
Birds health status			
Excellent	8	53.3	
Good	7	46.6	1.47±0.133[a]
Poor	0	0.0	
Mortality			
<1%	5	33.3	
1-2%	7	46.6	1.87±0.165[a]
>2%	3	20.0	
Antibiotic use			
Therapeutic purpose	4	26.6	
Prophylactic purpose	9	60.0	1.87±0.165[a]
Both	2	13.3	
Follow withdrawal period			
Yes	10	66.0	1.33±0.126[a]
No	5	33.3	

[a] indicated p>0.05 when compared with the characteristics in the same column, [b] indicated p<0.05 when compared with the characteristics parameter in the same column, [c] indicated p<0.01 when compared with the characteristics in the same column. SEM= Standard error of mean.

Supplementary Table 4. General characteristics of Duck farms in Noakhali region of Bangladesh

Characteristics	No of farms	%	Mean ± SEM
Duck farm (n=8)			
No. of birds			
500-1000	1	12.5	
1000-2000	5	62.5	2.12± 0.227[a]
>2000	2	25.0	
Breeding system			
All-in All out	8	100	1.00±0.00
Multi-age	0	0.0	
Types of feed			
Starter	1	12.5	
Grower	3	37.5	2.88±0.441[a]
Finisher	0	0.0	
Homemade	4	50.0	
Use of feed additive			
Yes	2	25.0	1.75±0.164[a]
No	6	75.0	
Presence of diarrhea			
Yes	2	25.0	1.75±0.164[a]
No	6	75.0	
Birds health status			
Excellent	5	62.5	
Good	3	37.5	1.38±0.183[a]
Poor	0	0.0	
Egg production performance			
Not yet	1	12.5	
Normal	5	62.5	2.12±0.227[a]
Decrease	2	25.0	
Egg type			
Normal	5	62.5	1.38±0.183[a]
Abnormal	3	37.5	
Mortality			
<1%	3	37.5	
1-2%	4	50.0	1.75±0.250[a]
>2%	1	12.5	
Antibiotic use			
Therapeutic purpose	2	25.0	
Prophylactic purpose	5	62.5	1.88±0.227[a]
Both	1	12.5	
Follow withdrawal period			
Yes	5	62.5	1.38±0.183[b]
No	3	37.5	

[a] indicated p>0.05 when compared with the characteristics in the same column,. SEM= Standard error of mean.

,

Supplementary Table 5. Biochemical characteristics of *Bacillus* spp.

Test	Bacterial species						
	B. cereus	*B. thuringiensis*	*B. amyloliquefaciens*	*B. subtilis*	*B. licheniformis*	*B. megaterium*	*B. coagulans*
Gram stain	+	+	+	+	+	+	+
Catalase	+	+	+	+	+	+	+
Egg yolk reaction	+	+	-	-	+	-	-
Motility	+	+	+	-	-	-	-
Mannitol	-	-	+	+	+	+	-
Citrate	+	+	+	+	+	+	-
VP	+	+	+	+	+	-	-
Nitrate reduction	+	+	+	+	+	-	-
Indole	-	-	-	-	-	-	-
Starch hydrolysis	+	+	+	+	+	+	+
Acid from							
Glucose	+	+	+	+	+	+	+
Lactose	-	-	+	+	+	+	+
Sucrose	-	-	+	+	+	+	+
Oxidase	-	-	+	+	-	-	-
Anaerobic growth	+	+	-	-	+	-	+

Supplementary Table 6. Prevalence of enterotoxin and emetic toxin gene of isolated *Bacillus* spp.

Sample types	Number of positive samples for enterotoxin and emetic toxins gene (%)									
	nheA	***nheB***	***nheC***	***cytk***	***hblA***	***hblC***	***hblD***	***entFM***	***Ces***	***All 8 genes***
LF (n=42)	31 (73.8)	35 (83.3)	31 (73.8)	32 (76.1)	18 (42.8)	19 (45.2)	18 (42.8)	32 (76.1)	0 (0.0)	13 (30.9)
BF (n=37)	29 (78.3)	34 (91.8)	30 (81.0)	31 (83.7)	26 (70.2)	20 (54.0)	21 (56.7)	29 (78.3)	0 (0.0)	15 (40.5)
DF (n=26)	20 (76.9)	24 (85.7)	20 (76.9)	17 (65.3)	15 (57.6)	13 (50.0)	14 (53.8)	24 (92.3)	0 (0.0)	9 (34.6)
CF (n=28)	23 (82.1)	24 (85.7)	19 (67.8)	24 (85.7)	18 (64.2)	10 (35.7)	14 (50.0)	25 (89.2)	0 (0.0)	7 (25.0)
FF (n=19)	15 (78.9)	15 (78.9)	14 (73.6)	14 (73.6)	12 (63.1)	8 (42.1)	11 (57.8)	16 (84.2)	0 (0.0)	5 (26.3)
E (n=25)	11 (44.0)	11 (44.0)	10 (40.0)	10 (40.0)	9 (36.0)	6 (24.0)	5 (20.0)	16 (64.0)	0 (0.0)	2 (8.0)
M (n=31)	20 (64.5)	17 (54.8)	15 (48.3)	20 (64.5)	19 (61.2)	5 (16.1)	16 (51.6)	24 (77.4)	0 (0.0)	1 (3.2)
HS (n=10)	7 (70.0)	8 (80.0)	6 (60.0)	7 (70.0)	8 (80.0)	6 (60.0)	7 (70.0)	9 (90.0)	0 (0.0)	5 (50.0)
Total= 218	156 (71.5)	168 (77.0)	145 (66.5)	155 (71.1)	125 (57.3)	87 (39.9)	106 (48.6)	175 (80.2)	0 (0.0)	57 (26.1)

LF=Layer feed, BF=Broiler feed, DF=Duck feed, CF=Cattle feed, FF=Fish feed, E=Egg, M=Milk, HS=Human stool

Supplementary Table 7. Prevalence of NHE and HBL gene complex of isolated *Bacillus* spp.

Sample types	Number of positive samples for NHE and HBL complex gene (%)									
	nheAB	***nheBC***	***nheAC***	***nheABC***	***nhe-ve***	***hblAC***	***hblCD***	***hblAD***	***hblACD***	***hbl-ve***
LF (n=42)	31 (73.8)	31 (73.8)	30 (71.4)	30 (71.4)	7 (16.6)	16 (38.0)	16 (38.0)	17 (40.4)	16 (38.0)	20 (47.6)
BF (n=37)	29 (78.3)	30 (81.0)	28 (75.6)	28 (75.6)	3 (8.1)	20 (54.0)	19 (51.3)	21 (56.7)	19 (51.3)	11 (29.7)
DF (n=26)	19 (73.0)	17 (65.3)	18 (69.9)	17 (65.3)	3 (11.5)	10 (38.4)	9 (34.6)	12 (46.1)	9 (34.6)	6 (23.0)
CF (n=28)	21 (75.0)	17 (60.7)	18 (64.2)	16 (57.1)	2 (7.1)	10 (35.7)	8 (28.5)	13 (46.4)	8 (28.5)	9 (32.1)
FF (n=19)	11 (57.8)	10 (52.6)	13 (68.4)	10 (52.6)	2 (10.5)	8 (42.1)	6 (31.5)	9 (47.3)	6 (31.5)	5 (26.3)
E (n=25)	7 (28.0)	9 (36.0)	8 (32.0)	7 (28.0)	10 (40.0)	4 (16.0)	4 (16.0)	4 (16.0)	4 (16.0)	13 (52.0)
M (n=31)	15 (48.3)	9 (29.0)	13 (41.9)	8 (25.8)	10 (32.2)	5 (16.1)	5 (16.1)	14 (45.1)	5 (16.1)	11 (35.4)
HS (n=10)	7 (70.0)	6 (60.0)	6 (60.0)	6 (60.0)	2 (20.0)	6 (60.0)	6 (60.0)	7 (70.0)	6 (60.0)	2 (20.0)
Total= 218	140 (64.2)	129 (59.1)	134 (61.4)	122 (55.9)	39 (17.8)	79 (36.2)	73 (33.4)	97 (44.4)	73 (33.4)	77 (35.3)

LF=Layer feed, BF=Broiler feed, DF=Duck feed, CF=Cattle feed, FF=Fish feed, E=Egg, M=Milk, HS=Human stool

Supplementary Table 8. Distribution of toxin genes in *Bacillus* spp. from animal feed, food and diarrhea

Source	Species	Tested isolates	Toxin gene								
			nheA	***nheB***	***nheC***	***cytK***	***hblA***	***hblC***	***hblD***	***entFM***	***ces***
AF	*B. cereus*	75	55 (73.3)	42 (56.0)	26 (34.6)	54 (72.0)	41 (54.6)	27 (36.0)	35 (46.6)	63 (84.4)	ND
	B. subtilis	38	11 (28.9)	ND	ND	ND	ND	ND	ND	ND	ND
	B. amylolequefaciens	8	ND	ND	ND	ND	ND	ND	ND	ND	ND
	B. licheniformis	13	4 (30.7)	ND	2 (15.3)	5 (38.4)	7 (53.8)	ND	3 (23.0)	8 (61.5)	ND
	B. thuringiensis	6	2 (33.3)	2 (33.3)	2 (33.3)	4 (66.6)	3 (50.0)	2 (33.3)	2 (33.3)	4 (66.6)	ND
	B. megaterium	8	2 (25.0)	ND	1 (12.5)	4 (50.0)	1 (12.5)	ND	1 (12.5)	2 (25.0)	ND
	B. coagulans	4	2 (50.0)	ND	ND	3 (75.0)	ND	ND	ND	2 (50.0)	ND
ADF	*B. cereus*	30	19 (63.3)	11 (36.6)	8 (26.6)	20 (66.6)	14 (46.6)	12 (40.0)	15 (50.0)	24 (80.0)	ND
	B. subtilis	8	2 (25.0)	ND	ND	ND	ND	ND	ND	ND	ND
	B. amylolequefaciens	12	ND	ND	ND	ND	ND	ND	ND	ND	ND
	B. licheniformis	ND	ND	ND	ND	ND	ND	ND	ND	ND	ND
	B. thuringiensis	5	2 (40.0)	1 (20.0)	1 (20.0)	3 (60.0)	3 (60.0)	2 (40.0)	1 (20.0)	3 (60.0)	ND
	B. megaterium	ND	ND	ND	ND	ND	ND	ND	ND	ND	ND
	B. coagulans	1	1 (100.0)	ND	ND	1 (100.0)	ND	ND	ND	1 (100.0)	ND
D	*B. cereus*	7	5 (71.4)	3 (42.8)	2 (28.5)	6 (85.7)	4 (57.4)	3 (42.8)	2 (28.5)	5 (71.4)	ND
	B. subtilis	3	1 (33.3)	ND	ND	ND	ND	ND	1 (33.3)	ND	ND
	B. amylolequefaciens	ND	ND	ND	ND	ND	ND	ND	ND	ND	ND
	B. licheniformis	ND	ND	ND	ND	ND	ND	ND	ND	ND	ND
	B. thuringiensis	ND	ND	ND	ND	ND	ND	ND	ND	ND	ND
	B. megaterium	ND	ND	ND	ND	ND	ND	ND	ND	ND	ND
	B. coagulans	ND	ND	ND	ND	ND	ND	ND	ND	ND	ND

AF=Animal feed (Layer+Broiler+Duck+Cattel+Fish), AFF=Animal Derive Food (Egg+Milk), D= Diarrhea (Human stool), ND=Not detected

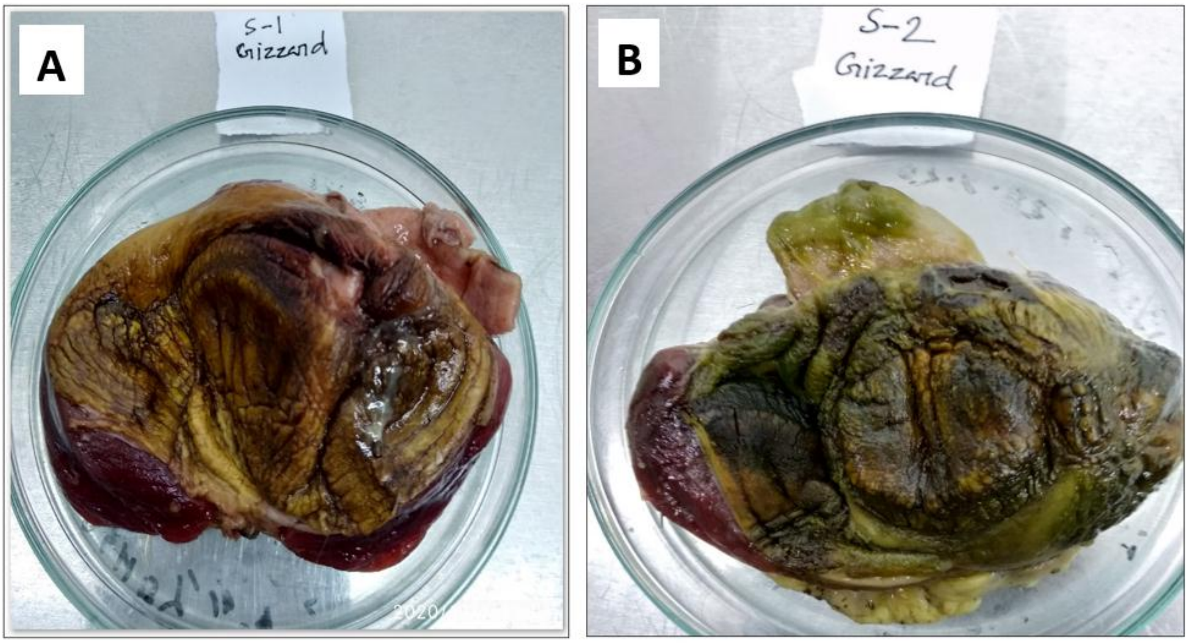

Supplementary Figure 1. A and B showing the gizzard erosion and ulceration of affected duck on postmortem examination during field outbreak.

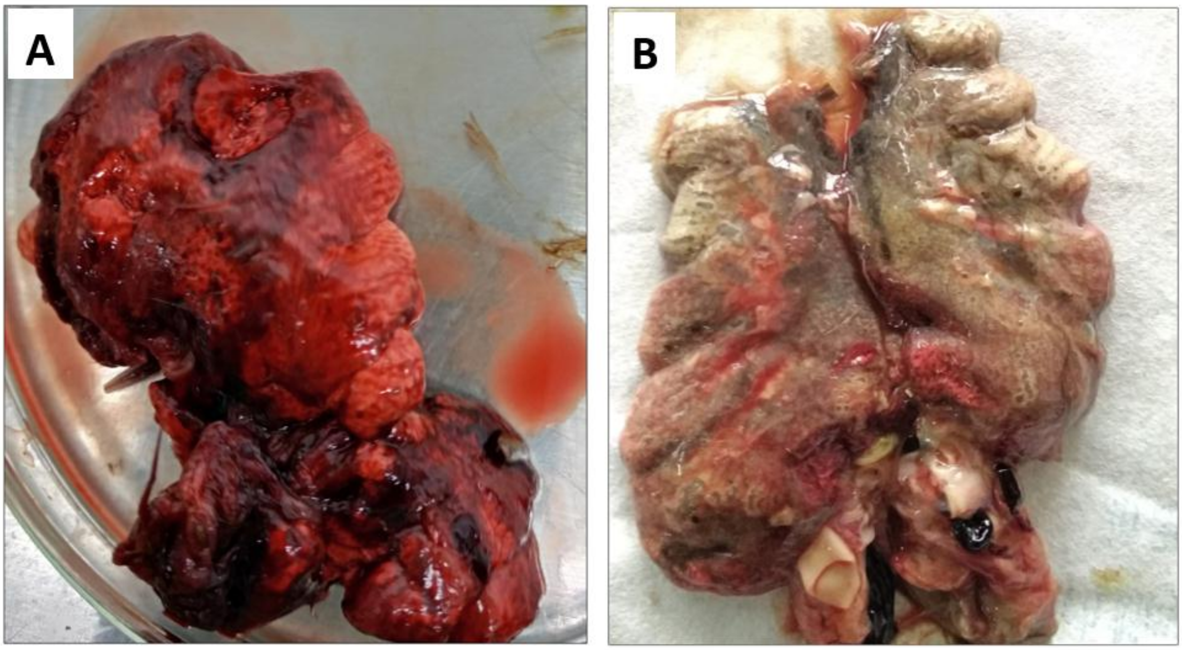

Supplementary Figure 2. A and B showing the severe hemorrhage and inflammation affected duck on postmortem examination during field outbreak.

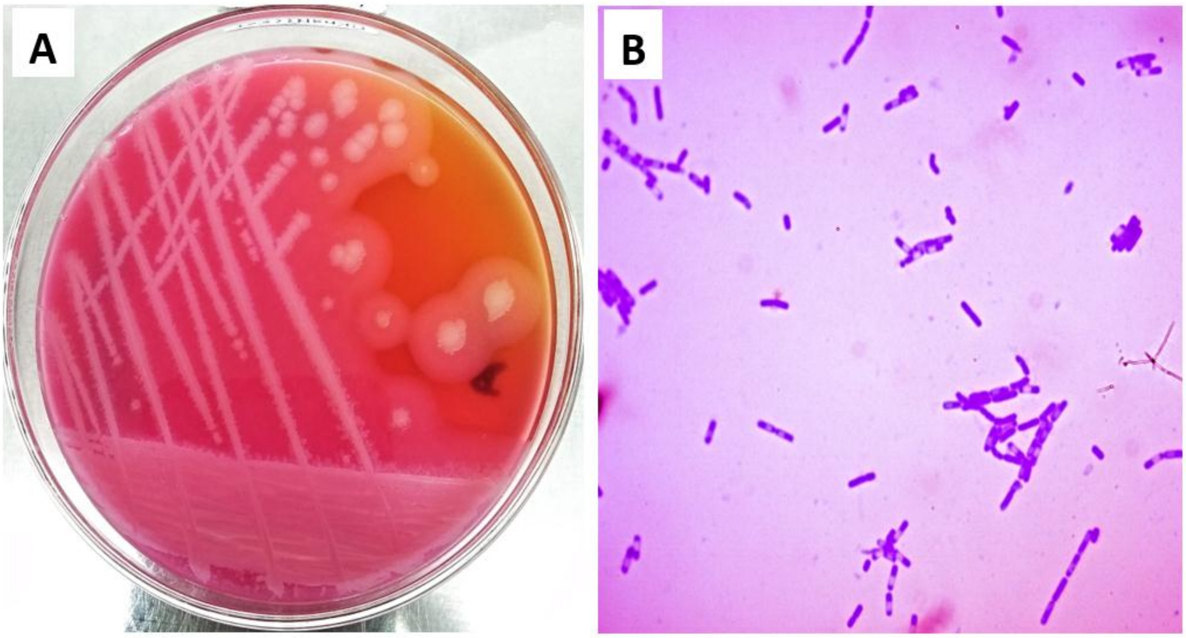

Supplementary Figure 3. A (Culture of isolated bacteria from gizzard and lungs of affected duck revealed *Bacillus cereus* on MYPA with characteristic egg yolk reaction with hollow precipitation zone); B (Gram staining showing gram positive rod shape with endospore arranged single or pair or forming chain).

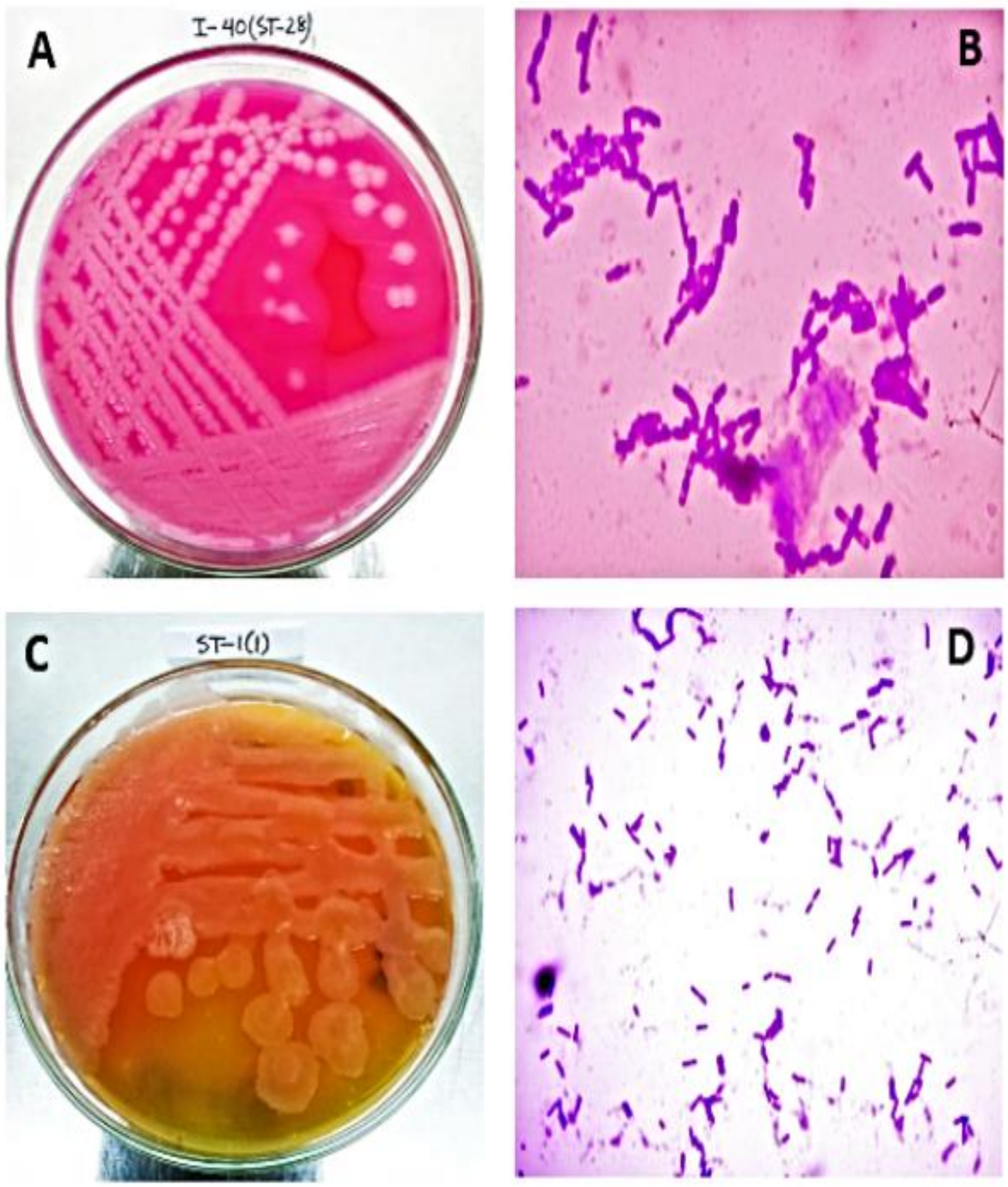

Supplementary Figure 4. A (Culture of isolated bacteria from human stool revealed *Bacillus cereus* on MYPA with characteristic egg yolk reaction with hollow precipitation zone) and B (Gram staining showing gram positive rod shape with endospore arranged single or pair or forming chain); C (Culture of isolated bacteria from human stool revealed *Bacillus subtilis* on MYPA showing mannitol positive revealing yellowish color of the medium) and D (Gram staining showing gram positive rod shape arranged single or pair or forming chain).

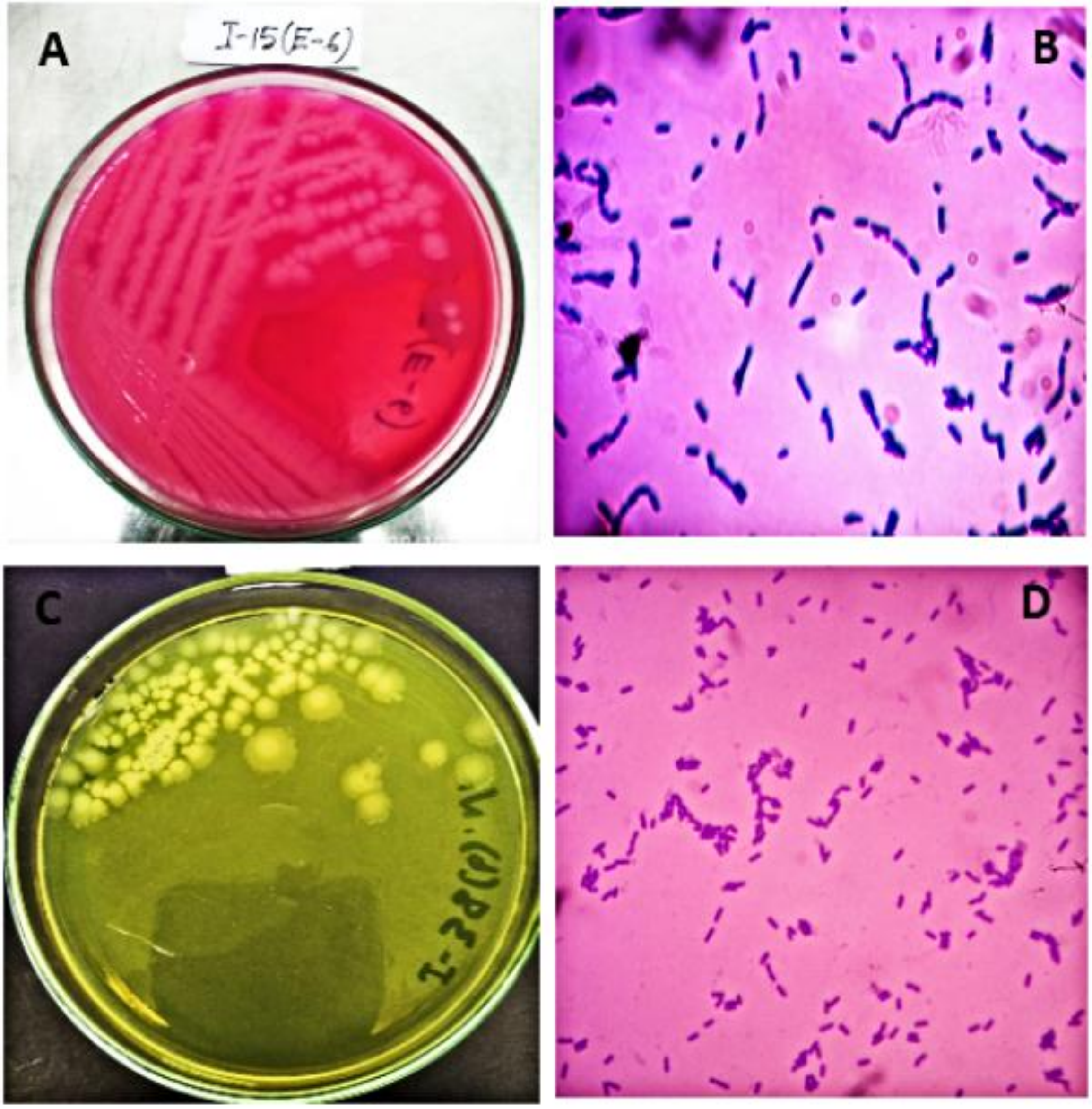

Supplementary Figure 5. **A (Culture of isolated bacteria from egg revealed *Bacillus thuringiensiss* on MYPA with characteristic egg yolk reaction with hollow precipitation zone) and B (Gram staining showing gram positive rod shape arranged single or pair or forming chain); C (Culture of isolated bacteria from egg revealed *Bacillus amyloliquefaciens* on NA showing characteristic colonies) and D (Gram staining showing gram positive rod shape arranged single or pair or forming chain).**
